# Early GnRH-agonist therapy does not negatively impact the endometrial repair process or live birth rate

**DOI:** 10.3389/fendo.2024.1343176

**Published:** 2024-04-29

**Authors:** Chen Wang, Yangqin Peng, Hui Chen, Qinmei Wang, Yu Dong, Huimin Liu, Yaoshan Yao, Shunji Zhang, Yuan Li, Sufen Cai, Xihong Li, Ge Lin, Fei Gong

**Affiliations:** ^1^ Institute of Reproductive and Stem Cell Engineering, NHC Key Laboratory of Human Stem Cell and Reproductive Engineering, School of Basic Medical Sciences, Central South University, Changsha, China; ^2^ Clinical Research Center for Reproduction and Genetics in Hunan Province, Reproductive and Genetic Hospital of CITIC-XIANGYA, Changsha, China; ^3^ Key Laboratory of Stem Cells and Reproductive Engineering, National Health and Family Planning Commission, Changsha, China

**Keywords:** intrauterine adhesion, adenomyosis, GnRH-a downregulation, frozen-thawed embryo transfer, clinical pregnancy, live birth

## Abstract

**Study objective:**

To investigate whether different timings of GnRH-a downregulation affected assisted reproductive outcomes in infertile women with moderate-to-severe intrauterine adhesions (IUAs) accompanied by adenomyosis.

**Design:**

A retrospective case series.

**Setting:**

An assisted reproductive technology center.

**Patients:**

The study reviewed 123 infertile women with moderate-to-severe IUAs accompanied by adenomyosis undergoing their first frozen-thawed embryo transfer (FET) cycles between January 2019 and December 2021.

**Measurements and main results:**

The majority of patients had moderate IUA (n=116, 94.31%). The average Basal uterine volume was 73.58 ± 36.50 cm^3^. The mean interval from operation to the first downregulation was 21.07 ± 18.02 days (range, 1–79 days). The mean duration of hormone replacement therapy (HRT) was 16.93 ± 6.29 days. The average endometrial thickness on the day before transfer was 10.83 ± 1.75 mm. A total of 70 women achieved clinical pregnancy (56.91%). Perinatal outcomes included live birth (n=47, 67.14%), early miscarriage (n=18, 25.71%), and late miscarriage (n=5, 7.14%). The time interval between uterine operation and the first downregulation was not a significant variable affecting live birth. Maternal age was the only risk factor associated with live birth (OR:0.89; 95% CI: 0.79–0.99, P=0.041).

**Conclusions:**

The earlier initiation of GnRH-a to suppress adenomyosis prior to endometrial preparation for frozen embryo transfer did not negatively impact repair of the endometrium after resection.

## Introduction

Intrauterine adhesions (IUAs) refer to a condition characterized by the partial or complete obstruction of the uterine cavity, which can arise due to aberrant healing processes subsequent to endometrial injury; complications may include dysmenorrhea, amenorrhea, infertility and recurrent miscarriages (1). IUAs can be effectively treated with hysteroscopic adhesiolysis, which releases adhesions and restores the cavity’s shape (1). Postoperative estrogen therapy is additionally recommended to promote endometrial repair and reduce adhesion reformation (2).

Adenomyosis is characterized by endometrial glands and stromal cells that invade the myometrium (3). The development of adenomyosis has been proven to be associated with increased estrogen exposure, multiparity, and endometrial injury. The rate of infertility among patients with adenomyosis is approximately 22% (4).Gonadotropin-releasing hormone agonists (GnRH-a) are widely used in adenomyosis therapy, and not only do they exert direct anti-proliferative effects on lesions, but they can also precipitate a hypoestrogenic state via competitive downregulation of pituitary GnRH receptors (GnRHRs) to treat adenomyosis (5). The use of GnRH-a before frozen embryo transfer (FET) has been associated with increased rates of clinical pregnancy in patients with adenomyosis (6).

As the pathogenesis of both IUAs and adenomyosis is related to uterine injury (1, 3), the incidence of IUAs accompanied by adenomyosis has been reported to be 17.2% (7). There are currently no guidelines for a treatment protocol of this combined pathology. Therefore, the objective of this study is to summarize the IVF/ICSI-FET procedure and reproductive outcomes of patients with the concurrence of these two disorders in our assisted reproduction center, and to investigate whether different timings of GnRH-a downregulation affected assisted reproductive outcomes.

## Materials and methods

### Study design

This was a retrospective case series. Patients with IUAs accompanied by adenomyosis who received IVF/ICSI-FET between January 2019 and December 2021 at the Reproductive and Genetic Hospital of CITIC-Xiangya were enrolled in this study. The analytical data were collected from medical records and telephone follow-up until November of 2022. The study was approved by the Ethics Committee of the Reproductive and Genetic Hospital of CITIC-Xiangya (Number LL-SC-2022-040).

### Patients

The inclusion criteria included patients who diagnosed moderate to severe IUAs at the first time (i.e., with an American Fertility Society (AFS) score ≥5) (8), patients diagnosed with adenomyosis (following the Morphological Uterus Sonographic Assessment (MUSA) criteria) (9), and the use of GnRH-a to treat adenomyosis after hysteroscopic adhesiolysis. The exclusion criteria included maternal age >40 years, downregulation more than six times, endometrial tuberculosis, a history of recurrent implantation failure, a history of recurrent spontaneous abortion, uterine intramural fibroids >2 cm, abnormal uterine anatomy, use of a levonorgestrel intrauterine device, and failure to restore normal uterine cavity shape after surgery.

### Intrauterine adhesiolysis and postoperative management

Patients received general intravenous anesthesia with propofol. Surgery was guided by transabdominal ultrasonography at the ovarian follicular stage. Normal saline was used as a distension medium, administered by a swelling pump with a flow rate of 280 mL/min, and the dilation pressure was set to 120 mmHg. Fluid monitoring was carried out by calculating the amount of liquid in a 5000-mL measuring cup, which collected the liquid flowing from the under buttocks drape (craniotomy incise drape, Jiangxi 3L Medical Products Group Co., Ltd., China). In accordance with the size of the cavity, type of IUA, and menstrual pattern, a quantitative severity score was designed: mild was indicated by 1-4, moderate was indicated by 5-8, and 9-12 indicated severe. The adhesions were dissected using bipolar energy (Olympus) and/or hysteroscopic scissors (Karl Storz, Tuttlingen, Germany) until the uterine cavity was achieved.

After surgery, a heart-shaped intrauterine balloon (Cook Medical, Bloomington, IN, USA) or Foley catheter was inserted into the uterine cavity as appropriate, depending on the patient’s uterine width. The average width of the uterus in primipara was reported as 27 mm (10, 11), and the minimal width of a COOK balloon was 28 mm. To avoid endometrial pressure (12), a heart-shaped intrauterine COOK balloon was only placed in patients with a uterine width of ≥28 mm, and they remained in place uninflated for at least one month. For patients with a uterine width <28 mm, Foley catheters were inflated with 2 mL of physiologic saline in case of fall-off and combined with early second-look hysteroscopy to prevent adhesion reformation (13). All patients received crosslinked hyaluronan gel (MateRegen; BioRegen Biomedical, Changzhou, China). In addition, all patients received standardized postoperative antibiotic therapy of 0.25 g of oral cefuroxime axetil and 0.5 g of tinidazole, both twice daily, for 7 days starting on the first postoperative day. None of the patients received HRT for the purposes of endometrial healing following the operation.

### GnRH-a downregulation and endometrial preparation procedures

The diagnosis of adenomyosis followed the MUSA criteria (9). Eight separate sonographic findings were used to identify the presumed adenomyosis: (a) asymmetric thickening, (b) cysts, (c) fan-shaped shadowing, (d) translesional vascularity, (e) echogenic subendometrial lines and buds, (f) hyperechoic islands, (g-h) irregular and interrupted junctional zone.

GnRH-a combined with HRT were used for endometrial preparation before frozen embryo transfer (FET). A long-acting GnRH-a (triptorelin; Ferring GmbH, Kiel, Germany) was administered at a dose of 3.75 mg every 28 days at least once (range, 1–6 times) starting in the early follicular stage or mid-luteal phase of the menstrual cycle (or the next menstrual cycle). The number of downregulation cycles depended on uterine size and the therapeutic effect achieved. One cycle was given if the uterus diameter (long diameter + wide diameter + anteroposterior diameter) was less than or equal to 150 mm, while two to three cycles were given for a diameter over 150 mm. An additional GnRH-a injection was given if the size of the adenomyosis lesion had not decreased. Serum was assayed for estradiol (E_2_), luteinizing hormone (LH), and human chorionic gonadotropin (HCG) to assess basal endocrine status and to exclude pregnancy before HRT was administered.

After the last dose of triptorelin was administered, HRT was started after 28 days. The HRT procedures we adopted were as described previously (14). estradiol valerate (2-6 mg orally daily) (Progynova, Delpharm Lille SAS, France) was administered for 10-15 days. When the endometrial thickness reached 8 mm, dydrogesterone (10mg orally 2 times daily) (Duphaston, Abbott Biologicals BV, The Netherlands) and progesterone medication utrogestan (200 mg vaginally three times daily) (Laboratoires Besins International, France) were administered. If a pregnancy has occurred, provide luteal phase support until 10 weeks of gestation.

### Embryo transfer procedure

Ovarian stimulation protocols, oocyte retrieval, IVF/ICSI, embryo vitrification freezing, and thawing procedures were performed as described in previous studies (15, 16). Cleavage-embryo transfer was performed on day 3 or blastocyst transfer on day 5 after progesterone administration. A maximum of two frozen-thawed cleavage-stage/blastocyst embryos were transferred.

### Data measurement method

The duration of HRT refers to the time from the use of estrogen to the transfer of the embryo. Classification at the cleavage stage was based on conventional criteria (17), and blastocyst quality assessment was based on the Gardner scoring system (18). Good quality embryos were defined as having a blastocyst rating of ≥4 BB or cleavage rating ≥7 CII. The formula of prolate ellipsoid volume was used to calculate the basal uterine volume: long diameter x wide diameter x anteroposterior diameter x0.523 (19). The endometrial thickness was measured the day before transfer, the number of abortions was defined as the number of previous induced and spontaneous abortions, clinical pregnancy was defined as the presence of an intrauterine gestational sac with fetal cardiac activity on transvaginal ultrasound four weeks after FET (excluding ectopic pregnancy), early miscarriage was defined as spontaneous loss of intrauterine pregnancy before 12 weeks of gestation, late miscarriage was defined as spontaneous loss of intrauterine pregnancy between 12 and 28 weeks of gestation, live birth was defined as a viable delivery beyond 28 weeks of gestation.

### Statistical analysis

The distribution of patient demographics and clinical characteristics was analyzed using the Kolmogorov–Smirnov test. Continuous variables were expressed as mean ± standard deviation (SD). Categorical variables were expressed as frequency and percentage. Univariate and multivariate logistic regression analyses were conducted to evaluate the possible influencing factors for a live birth outcome, and the odds ratios (ORs) and 95% confidence interval (CIs) were calculated. All the statistical analyses were conducted using SPSS 25.0 (Chicago, USA), and P<0.05 was considered statistically significant.

## Results

**Figure 1:**
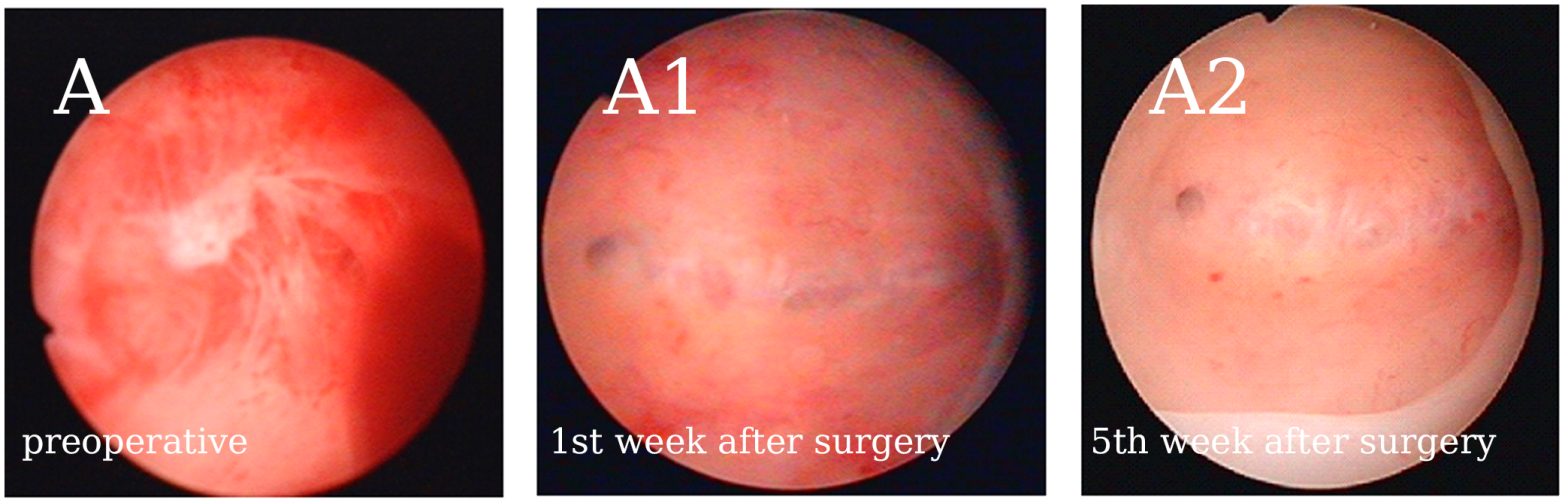
Representative continuous hysteroscopic images suggest that early GnRH-a downregulation after hysteroscopic adhesiolysis does not affect the normal endometrial repair process. **(A)** Patient no. 40’s AFS score was 10. **(A1)** There were no clear adhesions in the uterine cavity after cleaning the exudate the first week after surgery. **(A2)** No. 40 received GnRH-a downregulation once on the 9th day after surgery, and the endometrium showed good recovery in the 5fth week after surgery. We observed no adhesions or exudate, however, a thin endometrium was noted.

A total of 123 patients were included in the retrospective case series. **Figure 1** shows the continuous hysteroscopic images. None of our patients experienced uterine perforation, infection, excessive bleeding, water intoxication, or other complications during hysteroscopic adhesiolysis. Baseline characteristics are presented in **Table 1**. The average age was 33.20 ± 3.71 years (range, 21-40 years), and the average BMI was 22.54 ± 2.52 kg/m^2^. The majority of patients had secondary infertility (n=119, 96.75%). The average gravidity time was 2.54 ± 1.49, and the average number of previous induced and spontaneous abortions was 1.59 ± 1.17.

**Table 1:** Baseline characteristics.

Characteristic	Overall N =123^1^
Age (years)	33.20 ± 3.71
≤35 y	88/123 (71.54%)
>35 y	35/123 (28.46%)
BMI (kg/m^2^)	22.54 ± 2.52
Primary infertility	4/123 (3.25%)
Secondary infertility	119/123 (96.75%)
Gravidity	2.54 ± 1.49
Parity	0.34 ± 0.56
No. of previous induced and spontaneous abortions	1.59 ± 1.17

^1^Mean ± SD; n/N(%).

BMI, body mass index.

**Table 2** summarizes uterine conditions before frozen-thawed embryo transfer and reproductive outcomes. The average AFS score was 6.34 ± 1.38. The majority of patients had moderate IUA (n=116, 94.31%). Physical barrier types included a heart-shaped intrauterine balloon (n=89, 72.36%) and Foley catheter (n=34, 27.64%). The average basal uterine volume was 73.58 ± 36.50 cm^3^. The mean interval from the operation to the first downregulation was 21.07 ± 18.02 days (range, 1–79 days). The number of downregulations included one time (n=11, 8.94%), two times (n=87, 70.73%), and three or more times (n=25, 20.33%). Serum E_2_ (11.80 ± 10.03 pg/mL) and LH levels (0.54 ± 0.61 mIU/mL) in the 3 days before HRT indicated a state of pituitary downregulation. The mean duration of HRT was 16.93 ± 6.29 days.

**Table 2:** Uterine conditions and reproductive outcomes. .

Characteristic	Overall N =123^1^
Uterine conditions	
AFS score	6.34 ± 1.38
Grades by AFS score	
Moderate	116/123 (94.31%)
Severe	7/123 (5.69%)
Physical barrier types	
Heart-shaped intrauterine balloon	89/123 (72.36%)
Foley catheter	34/123 (27.64%)
Basal uterine volume (cm^3^)	73.58 ± 36.50
Time interval between operation and first downregulation (days)	21.07 ± 18.02
No. of downregulations	
1	11/123 (8.94%)
2	87/123 (70.73%)
≥3	25/123 (20.33%)
E2 levels in the three days before HRT (pg/ml)	11.80 ± 10.03
LH levels in the three days before HRT (mIU/ml)	0.54 ± 0.61
Duration of HRT (days)	16.93 ± 6.29
Endometrial thickness on the day before transplantation (mm)	10.83 ± 1.75
No. of embryos transferred	1.28 ± 0.45
No. of embryos transferred	
1	89/123 (72.36%)
2	34/123 (27.64%)
Embryo types	
Cleavage	18/123 (14.63%)
blastocyst	105/123 (85.37%)
Embryo quality	
At least one good-quality embryo	79/123 (64.22%)
Non-good-quality embryo	44/123 (35.77%)
Reproductive outcomes	
Clinical pregnancy	70/123 (56.91%)
Live-birth rate	47/70 (67.14%)
Early miscarriage	18/70 (25.71%)
Late miscarriage	5/70 (7.14%)

^1^Mean ± SD; n/N(%).

AFS, American Fertility Society; HRT, hormone replacement therapy; E2, estradiol; LH, luteinizing hormone.

None of the patients had their frozen-thawed embryo transfer cycle cancelled due to thin endometrium and/or adhesion reformation. The average endometrial thickness on the day before transfer was 10.83 ± 1.75 mm. A total of 70 women achieved clinical pregnancy (56.91%). Perinatal outcomes included live birth (n=47, 67.14%), early miscarriage (n=18, 25.71%), and late miscarriage (n=5, 7.14%). Among the 9 cases (50%) of early miscarriage, embryonic genetic testing was conducted, and in 6 cases (66.7%), chromosomal abnormalities were found.

Logistic regression analysis was conducted to determine the independent prognostic factors concerning the probability of live birth. We included all the variables in the regression analysis, and the statistically significant positive results are presented in **Table 3**. The time interval between uterine operation and the first downregulation was not a significant variable affecting live birth. Maternal age was the only risk factor associated with live birth (OR:0.89; 95% CI: 0.79–0.99, P=0.041).

**Table 3:** Univariate and multivariate analyses of pregnancy and live-birth outcome.

Characteristic	Live birth					
	Univariate analysis			Multivariate analysis		
	OR	95% CI	P	OR	95% CI	P
Age	0.87	0.79-0.97	**0.012**	0.89	0.79-0.99	**0.041**
BMI	1.02	0.88-1.18	0.811			
Infertility type (primary vs. secondary)	1.64	0.22-12.09	0.625			
Gravidity	0.8	0.61-1.04	0.093			
Parity	0.54	0.26-1.12	0.096			
No. of previous induced and spontaneous abortions	0.84	0.6-1.17	0.305			
AFS score	0.93	0.71-1.21	0.584			
Grades by AFS score (severe vs. moderate)	0.63	0.12-3.39	0.592	0.73	0.09, 4.26	0.737
Physical barrier (Foley catheter vs. Heart-shaped balloon)	1.19	0.53-2.66	0.676			
Basal uterine volume	0.99	0.98-1.00	**0.038**	1.00	0.98, 1.01	0.448
E2 levels in the three days before HRT (pg/ml)	1.01	0.97, 1.05	0.583			
LH levels in the three days before HRT (mIU/ml)	2.36	1.05, 7.43	0.093			
No. of downregulation	0.67	0.36, 1.11	0.155			
Two times vs.one time	4.8	1.03-22.37	**0.046**	0.70	0.16,3.00	0.623
Three or more times vs. one time	2.82	0.97-8.22	0.057	0.36	0.05, 2.26	0.277
Duration of HRT	1.02	0.96-1.07	0.607	1.01	0.94-1.07	0.861
Endometrial thickness on the day before transplantation	0.85	0.69-1.06	0.158			
No. of embryos transferred (2 vs. 1)	1.00	0.44, 2.24	0.997			
Embryo types (blastocyst vs. Cleavage)	3.61	1.11, 16.2	0.053			
Good-quality embryo (yes vs. no)	0.97	0.46-2.08	0.942			
Time interval between operation and first downregulation	1.01	0.99-1.03	0.532	1.01	0.98, 1.03	0.575

BMI, body mass index; AFS, American Fertility Society; E2, estradiol; LH, luteinizing hormone; ET, embryo transfer.

HRT, hormone replacement therapy.

p value < 0.05 was show in bold.

## Discussion

Endometrial repair after hysteroscopic adhesiolysis requires at least one month (20). However, the impact of early postoperative GnRH-a downregulation on endometrial repair remains controversial. Our preliminary findings suggest that the timing of GnRH-a administration (21.07 ± 18.02 days; range, 1–79 days), after hysteroscopic adhesiolysis, does not appear to affect the live-birth rate (**Table 3**). The durations of HRT (16.93 ± 6.29 days) and the endometrial thickness (10.83 ± 1.75 mm) also indicate that endometrial healing in a hypoestrogenic state does not decrease the reactivity to estrogen. Continuous hysteroscopic imaging demonstrates the normal endometrial repair process under early GnRH-a downregulation postoperative.

Although estrogen therapy is recommended after hysteroscopic adhesiolysis (2), its efficacy is debated. Several studies have indicated that estrogen supplementation after hysteroscopic surgery does not reduce the incidence of IUAs (21), nor does it increase pregnancy rates or reduce miscarriage rates (22, 23). The addition of estrogen after hysteroscopic adhesiolysis also does not reduce the re-adhesion rates in both mild and severe IUAs (24). In addition, higher estrogen doses did not improve postoperative outcomes. Liu L et al. illustrated there was no significant difference in AFS scores at second look hysteroscopy between the two doses of oestradiol valerate groups after hysteroscopic adhesiolysis (4 mg and 10 mg daily), nor in conception rate or miscarriage rate (25). Similar results have been found in another study (26). Consequently, estrogen therapy is not recommended as a routine postoperative treatment (24).

There are several possible interpretations of our results: (1) Estrogen may not be involved in endometrial repair, but rather in endometrial proliferation. Animal models of endometrial repair have shown that estrogen is not required for endometrial re-epithelialization (27, 28), and the endometrium can be repaired spontaneously in postmenopausal women and those who have undergone oophorectomy (29). Previous studies have also indicated that hysteroscopic surgery (with danazol pretreatment) in a low-estrogen state does not increase the incidence of postoperative uterine adhesions, aligning with our results (30). (2) All patients received a uterine barrier of some sort: either a Cook balloon or Foley catheter. This preventative measure may likely be the reason for the lack of adhesion reformation. It is possible that the early initiation of GnRH-a therapy would have a detrimental impact on endometrial repair if the adhesion barrier is not utilized.

There were some limitations to this retrospective cohort study. We were not able to collect data on uterine volume after GnRH-a treatment due to the fact that ultrasonographic assessments are not routinely recommended. Another possible criticism of our study is the retrospective case series, further research, especially in the form of cohort studies, is required to investigate the appropriate time for downregulation in infertile women with moderate to severe IUAs accompanied by adenomyosis.

## Conclusions

We herein demonstrated that, for infertile women with moderate to severe IUAs accompanied by adenomyosis, GnRH-a downregulation, whether administered sooner or later after hysteroscopic adhesiolysis, was not a significant variable affecting live birth. Moreover, this regimen could achieve satisfactory endometrial thickness under HRT.

## Data availability statement

The raw data supporting the conclusions of this article will be made available by the authors, without undue reservation.

## Ethics statement

The study was approved by the Ethics Committee of the Reproductive and Genetic Hospital of CITIC-Xiangya (Number LL-SC-2022-040). The studies were conducted in accordance with the local legislation and institutional requirements. Written informed consent for participation was not required from the participants or the participants’ legal guardians/next of kin in accordance with the national legislation and institutional requirements. Written informed consent was obtained from the individual(s) for the publication of any potentially identifiable images or data included in this article.

## Author contributions

CW: Conceptualization, Data curation, Writing – original draft, Writing – review & editing. YP: Formal analysis, Methodology, Writing – review & editing. HC: Investigation, Visualization, Writing – review & editing. QW: Data curation, Writing – review & editing. YD: Writing – review & editing. HL: Writing – review & editing. YY: Writing – review & editing. SZ: Writing – review & editing. YL: Writing – review & editing. SC: Writing – review & editing. XL: Writing – review & editing. GL: Writing – review & editing. FG: Conceptualization, Investigation, Project administration, Supervision, Writing – review & editing.

## References

[1] Yu D Wong YM Cheong Y Xia E Li TC. Asherman syndrome–one century later. Fertil Steril. (2008) 89(4):759–79. doi: 10.1016/j.fertnstert.2008.02.096 18406834

[2] AAGL practice report: practice guidelines for management of intrauterine synechiae. J Minim Invasive Gynecol. (2010) 17(1):1–7. doi: 10.1016/j.jmig.2009.10.009 20129325

[3] Bulun SE Yildiz S Adli M Wei JJ. Adenomyosis pathogenesis: insights from next-generation sequencing. Hum Reprod Update. (2021) 27:1086–97. doi: 10.1093/humupd/dmab017 PMC8543024 34131719

[4] Puente JM Fabris A Patel J Patel A Cerrillo M Requena A. Adenomyosis in infertile women: prevalence and the role of 3D ultrasound as a marker of severity of the disease. Reprod Biol Endocrinol. (2016) 14(1):60. doi: 10.1186/s12958-016-0185-6 27645154 PMC5029059

[5] Khan KN Kitajima M Hiraki K Fujishita A Nakashima M Ishimaru T. Cell proliferation effect of GnRH agonist on pathological lesions of women with endometriosis, adenomyosis and uterine myoma. Hum Reprod. (2010) 25:2878–90. doi: 10.1093/humrep/deq240 20829343

[6] Park CW Choi MH Yang KM Song IO. Pregnancy rate in women with adenomyosis undergoing fresh or frozen embryo transfer cycles following gonadotropin-releasing hormone agonist treatment. Clin Exp Reprod Med. (2016) 43:169–73. doi: 10.5653/cerm.2016.43.3.169 PMC5039310 27689040

[7] Wang J Movilla P Chen T Wang J Morales B Williams A. Concomitant adenomyosis among patients with asherman syndrome. J Minim Invasive Gynecol. (2021) 28:358–365.e1. doi: 10.1016/j.jmig.2020.07.011 32712321

[8] The American Fertility Society classifications of adnexal adhesions, distal tubal occlusion, tubal occlusion secondary to tubal ligation, tubal pregnancies, müllerian anomalies and intrauterine adhesions. Fertil Steril. (1988) 49(6):944–55. doi: 10.1016/S0015-0282(16)59942-7 3371491

[9] Van den Bosch T Dueholm M Leone FP Valentin L Rasmussen CK Votino A. Terms, definitions and measurements to describe sonographic features of myometrium and uterine masses: a consensus opinion from the Morphological Uterus Sonographic Assessment (MUSA) group. Ultrasound Obstet Gynecol. (2015) 46:284–98. doi: 10.1002/uog.14806 25652685

[10] Benacerraf BR Shipp TD Lyons JG Bromley B. Width of the normal uterine cavity in premenopausal women and effect of parity. Obstet Gynecol. (2010) 116:305–10. doi: 10.1097/AOG.0b013e3181e6cc10 20664389

[11] Wildemeersch D Hasskamp T Nolte K Jandi S Pett A Linden S. A multicenter study assessing uterine cavity width in over 400 nulliparous women seeking IUD insertion using 2D and 3D sonography. Eur J Obstet Gynecol Reprod Biol. (2016) 206:232–8. doi: 10.1016/j.ejogrb.2016.09.023 27768966

[12] Shipp TD Bromley B Benacerraf BR. The width of the uterine cavity is narrower in patients with an embedded intrauterine device (IUD) compared to a normally positioned IUD. J Ultrasound Med. (2010) 29:1453–6. doi: 10.7863/jum.2010.29.10.1453 20876899

[13] Pabuccu R Onalan G Kaya C Selam B Ceyhan T Ornek T. Efficiency and pregnancy outcome of serial intrauterine device-guided hysteroscopic adhesiolysis of intrauterine synechiae. Fertil Steril. (2008) 90:1973–7. doi: 10.1016/j.fertnstert.2007.06.074 18774563

[14] Gan RX Li Y Song J Wen Q Lu GX Lin G. Pregnancy outcomes of different endometrial preparation in patients with a history of cesarean section. Front Endocrinol (Lausanne). (2022) 13:813791. doi: 10.3389/fendo.2022.813791 35846338 PMC9280671

[15] Li Y Wen Q Liao J Ma S Zhang S Gu Y. Trophectoderm biopsy differentially influences the level of serum β-human chorionic gonadotropin with different embryonic trophectoderm scores in early pregnancy from 7847 single-blastocyst transfer cycles. Front Endocrinol (Lausanne). (2022) 13:794720. doi: 10.3389/fendo.2022.794720 35250858 PMC8894721

[16] Tan YQ Tan K Zhang SP Gong F Cheng DH Xiong B. Single-nucleotide polymorphism microarray-based preimplantation genetic diagnosis is likely to improve the clinical outcome for translocation carriers. Hum Reprod. (2013) 28:2581–92. doi: 10.1093/humrep/det271 23847111

[17] Istanbul consensus workshop on embryo assessment: proceedings of an expert meeting. Reprod BioMed. (2011) 22(6):632–46. doi: 10.1016/j.rbmo.2011.02.001 21481639

[18] Schoolcraft WB Gardner DK Lane M Schlenker T Hamilton F Meldrum DR. Blastocyst culture and transfer: analysis of results and parameters affecting outcome in two in *vitro* fertilization programs. Fertil Steril. (1999) 72:604–9. doi: 10.1016/S0015-0282(99)00311-8 10521095

[19] O’Donnell RL Warner P Lee RJ Walker J Bath LE Kelnar CJ. Physiological sex steroid replacement in premature ovarian failure: randomized crossover trial of effect on uterine volume, endometrial thickness and blood flow, compared with a standard regimen. Hum Reprod. (2012) 27(4):1130–8. doi: 10.1093/humrep/des004 22343553

[20] Yang JH Chen MJ Chen CD Chen SU Ho HN Yang YS. Optimal waiting period for subsequent fertility treatment after various hysteroscopic surgeries. Fertil Steril. (2013) 99(7):2092–6.e3. doi: 10.1016/j.fertnstert.2013.01.137 23433831

[21] Healy MW Schexnayder B Connell MT Terry N DeCherney AH Csokmay JM. Intrauterine adhesion prevention after hysteroscopy: a systematic review and meta-analysis. Am J Obstet Gynecol. (2016) 215(3):267–275.e7. doi: 10.1016/j.ajog.2016.05.001 27173082

[22] Roy KK Negi N Subbaiah M Kumar S Sharma JB Singh N. Effectiveness of estrogen in the prevention of intrauterine adhesions after hysteroscopic septal resection: a prospective, randomized study. J Obstet Gynaecol Res. (2014) 40(4):1085–8. doi: 10.1111/jog.12297 24612233

[23] Tonguc EA Var T Yilmaz N Batioglu S. Intrauterine device or estrogen treatment after hysteroscopic uterine septum resection. Int J Gynaecol Obstet. (2010) 109:226–9. doi: 10.1016/j.ijgo.2009.12.015 20152976

[24] Yang L Ma N Song D Huang X Zhou Q Guo Y. The effect of estrogen in the prevention of adhesion reformation after hysteroscopic adhesiolysis: A prospective randomized control trial. J Minim Invasive Gynecol. (2022) 29:871–8. doi: 10.1016/j.jmig.2022.04.004 35439645

[25] Liu L Huang X Xia E Zhang X Li TC Liu Y. A cohort study comparing 4 mg and 10 mg daily doses of postoperative oestradiol therapy to prevent adhesion reformation after hysteroscopic adhesiolysis. Hum Fertil (Camb). (2019) 22:191–7. doi: 10.1080/14647273.2018.1444798 29504823

[26] Guo J Li TC Liu Y Xia E Xiao Y Zhou F. A prospective, randomized, controlled trial comparing two doses of oestrogen therapy after hysteroscopic adhesiolysis to prevent intrauterine adhesion recurrence. Reprod BioMed. (2017) 35:555–61. doi: 10.1016/j.rbmo.2017.07.011 28784336

[27] Matsuura-Sawada R Murakami T Ozawa Y Nabeshima H Akahira J Sato Y. Reproduction of menstrual changes in transplanted human endometrial tissue in immunodeficient mice. Hum Reprod. (2005) 20:1477–84. doi: 10.1093/humrep/deh783 15734760

[28] Kaitu’u-Lino TJ Morison NB Salamonsen LA. Estrogen is not essential for full endometrial restoration after breakdown: lessons from a mouse model. Endocrinology. (2007) 148:5105–11. doi: 10.1210/en.2007-0716 17640986

[29] Evans J Salamonsen LA Winship A Menkhorst E Nie G Gargett CE. Fertile ground: human endometrial programming and lessons in health and disease. Nat Rev Endocrinol. (2016) 12:654–67. doi: 10.1038/nrendo.2016.116 27448058

[30] Taskin O Sadik S Onoglu A Gokdeniz R Erturan E Burak F. Role of endometrial suppression on the frequency of intrauterine adhesions after resectoscopic surgery. J Am Assoc Gynecol Laparosc. (2000) 7:351–4. doi: 10.1016/S1074-3804(05)60478-1 10924629

